# Assessment of procrastination in providing nursing care among Iranian nursing staff

**DOI:** 10.1186/s12912-022-01132-5

**Published:** 2022-12-05

**Authors:** Mohadese Babaie, Azam Shirinabadi Farahani, Manijeh Nourian, Mahdi Hosseini, Arman Mohammadi

**Affiliations:** 1 Department of Pediatric Nursing, School of Nursing and Midwifery, Shahid Beheshti University of Medical Sciences, Tehran, Iran; 2 Tehran Heart Center, Tehran University of Medical Sciences, Tehran, Iran; 3 Nursing Care Research Center (NCRC), School of Nursing and Midwifery, Iran University of Medical Sciences, Tehran, Iran

**Keywords:** Procrastination, Nurses, Delay in nursing care, Intensive care units, Surgery wards

## Abstract

**Aim:**

This study aimed to investigate procrastination in nursing care providing.

**Design:**

This descriptive cross-sectional study was carried out on 125 nurses in ICUs, PICUs, NICUs, and surgery wards, who were selected by census sampling in Iran.

**Methods:**

The data were collected using the Procrastination Scale, which consisted of 25 items relating to 3 factors. Data were analyzed using statistics, Chi-square, Friedman test, analysis of variance, and Kolmogorov–Smirnov tests.

**Results:**

Overall, 37% of the participants showed very high or high procrastination. Most of the procrastination was observed in the “Task aversion” (44.2%). ANOVA indicated that the mean total procrastination score had a significant relationship with age (*p* = 0.013), work experience (*p* = 0.006), and marital status (*p* = 0.02). Nurses with permanent employment (*p* = 0.014) and lower education (*p* = 0.009) and women (*p* = 0.023) were much more likely to procrastinate the provision of care.

**Conclusion:**

It is recommended to adopt appropriate management strategies and take adequate measures to reduce procrastination, considering the existence of procrastination among nurses and its adverse impact on the quality of care.

## Introduction

An organization to achieve the objectives and the highest level of productivity, despite the availability of financial resources and new technology, is doomed to failure if it lacks a motivated workforce [1]. Nurses are considered vital workers in the healthcare system [2] and influential individuals in multidisciplinary healthcare teams who have an essential role in patient-centered care and health promotion, so the quality of their care and treatment is crucial. One of the issues of interest in quality and safe nursing care is ensuring the use of competent, regulated, and motivated staff in different parts of the health care system [3], and providing high-quality care is their responsibility as an organizational objective [4]. The nursing profession is considered a stressful job that faces different challenges in the workplace [5], such as procrastination, affecting the performance of nurses, particularly in surgery and intensive care units (ICUs), which provide emergency care.

## Background

Procrastination, intentionally delaying without any reason [6], is a kind of inability or lack of self-regulation in the form of a lack of control of thoughts, emotions, and motivation for professional performance, time management, organization, and inspiration for achievement, which can cause distress and reduce the sense of well-being [7, 8]. It is not only an expected delay in better decision-making but also a characteristic feature that leads to a delay in starting tasks and a loss of opportunities [9]. Procrastination affects performing duties in personal life, finance, investment, and health and has some negative consequences, such as mental health issues, depression, anxiety, low self-efficiency, and delays in work [10].

Various factors are related to individual characteristics, tasks, and the interaction between them for procrastination [9, 11, 12]. Task aversion refers to the nature of the job and rewards and penalties in the workplace. The employees are affected by personality traits such as boredom or lack of an inherent motivation to work. The nature of the task, in turn, causes disgust and is more likely to be procrastinated [13]. Mental anxiety, as a psychosocial factor, is another element that has proven to be related to procrastination. This state is a feeling of tension and apprehension or intrapsychic conflict. In other words, procrastination is related to worrying thoughts such as fear of failure and lack of success at work, social anxiety, depression, and the adverse consequences of mental anxiety [11].

In addition, procrastination can be an incorrect behavioral habit that ultimately decreases a person's self-efficiency. In this point of view, procrastination is a personality disorder known as a weakness of conscience with characteristics such as a lack of willpower, perseverance, attention, a weak desire for power, and inefficiency [14, 15]. Overall, delays in starting tasks result from failure to identify or lack of control that causes distress, guilt, loss of personal productivity and social disapproval, and suffering [7]. When these feelings are mixed, delays in starting tasks increase and may affect the natural functions of the individual. Therefore, this feature requires precise targeting to solve the problem [16].

The literature shows that most studies have investigated the amount of procrastination among students [17, 18], especially in the medical sciences. The results exponent the relationship between the tendency of procrastination with psychosocial factors, demographic, and other predictive variables [11, 19–21]. Also, several studies in this field investigated the effectiveness of psychological treatments [14].

The studies conducted to investigate this vital issue among nursing staff are limited [22]. On the other hand, in intensive care units, hospitalized patients often have critical and threatening conditions and require various professional care [23, 24]. Therefore, not neglecting to perform nursing duties is a crucial issue in care. In these units, procrastination can cause irreparable complications for patients.

Despite the importance of this issue, the number of staff who are postponing tasks is increasing [25]. Delays in nursing care, especially in high-risk wards such as surgery and intensive care units, are more consequential. It sometimes causes irreparable harm to patients and jeopardizes their safety [26]. While ensuring patient safety is guaranteed by the responsibility and commitment of nurses [23]. Consequently, timely care in critical care units, depending on the sensitivity of care and the specific situation of patients, is known as a professional challenge.

Consciousness and interventions based on this concept can reduce emotional exhaustion while maintaining mental health, lead to job satisfaction among nurses [27] and promote patient interaction and clinical performance. In this regard, this study was conducted to investigate procrastination in providing nursing care in surgery wards and intensive care units.

## Methods

### Study design

This cross-sectional descriptive quantitative design was used to assess procrastination in providing nursing care among Iranian nursing staff. This study was conducted through a census sampling of nurses who work in Educational Surgery and Intensive Care Units (NICUs, PICUs, SPICU, and ICUs) in Karaj, Iran, from November 2019 to late January 2020.

### Setting

This study was conducted in the two most prominent educational and treatment metropolitan hospitals, which include two large subspecialty and specialized hospitals concentrated in one complex (approximately 700 beds). In this complex, about 100 beds are allocated to several surgical and intensive care units of different sizes, which admit 35–150 patients per month in each unit. Of all staff, 160 nurses were working in these wards.

Neonates with congenital anomalies, acute respiratory problems at birth, and complications due to prematurity; pediatrics with medical and surgical diseases need invasive and critical care, such as cardiopulmonary disease or gastrointestinal and orthopedic surgeries admitted in these units. Also, adults are hospitalized with cardiopulmonary, nerve injuries, digestion, and urogenital system diseases.

### Participants and procedures

The population included nurses from surgery and intensive care units. The inclusion criteria for participants were all nurses who were at least in bachelor's degree in science, physical and psychological health (according to their reports and medical records), had different employment statuses, and had at least two years of work experience in these units. Those who were nursing managers (matrons and supervisors) were excluded.

First, a list of nurses was prepared. Out of 160 nurses on the list, 125 met the inclusion criteria. Since the sampling of this research was carried out in the two educational and treatment metropolitan hospitals in Karaj, Iran, where nurses working in NICUs, PICUs, ICUs, and surgery wards are limited, and considering the possibility of dropping samples, the participants were selected by census sampling. A census sampling is a collection of information from all population units. This method is chosen when we want detailed information for every population subset. Usually, such a survey requires a large sample size, and a census often provides the best solution. It also provides an accurate measure of the population (without sampling error), and benchmark data may be obtained for future studies.

The objectives and methods of the study were explained to the authorities. Informed consent was obtained before the participants filled out the questions. As a result, 125 nurses were examined. Five questionnaires were excluded from the analysis due to incomplete filling, and a total of 120 were analyzed.

### Instruments and measures

Data were collected using an organizational procrastination questionnaire developed based on the "Lay's General Procrastination Scale" [28, 29]. The first part of the questionnaire examines demographic information such as age, sex, marital status, education, work experience, and employment status. The second part assesses behavioral procrastination. It consists of 25 items, including three dimensions of important procrastination factors, as there were 16 questions in the "Inefficiency" dimension, five questions in the "Mental anxiety" dimension, and four questions in the "Task aversion" dimension. Grading was performed based on the five-point Likert scale: always = 5, often = 4, sometimes = 3, rarely = 2 and, 1 = never. Total scores vary from 25 to 125. The scores between 25 and 37 indicated very low procrastination or lack of procrastination; the scores between 113 and 125 showed high procrastination. The questionnaire was also used in domestic studies [22, 29]. In the present study, the nurses completed the questionnaire, which was in the Farsi language. It took about 10 min to complete the survey.

### Validity and reliability

Since the questionnaire was not previously used in the nurses’ population, its validity and reliability have been investigated. For assessing the qualitative content validity, the use of correct words, and the importance of the items [30], were judged by three faculty members and eight research experts, and amendments were made. For the qualitative face validity, the questionnaire was given to 10 selected nurses in a targeted manner, considering the diversity of work experience [31, 32].

The total reliability of the questionnaire was examined by Cronbach’s α internal consistency (0.84). Cronbach’s α was also acceptable for each dimension of the questionnaire, Inefficiency (0.83), Mental anxiety (0.78), and Task aversion (0.81).

### Data analysis

The data collected were analyzed in the SPSS software 22. The demographic characteristics are described using percent and frequency (descriptive statistics). Differences between the mean score in three dimensions of "Inefficiency", "Mental anxiety", and "Task aversion", and the level of procrastination were compared using the Friedman test and chi-square.

The analysis of variance (ANOVA) test is used to compare procrastination in different demographic characteristics. The normality of data distribution was assessed with Kolmogorov–Smirnov tests (standard setting). P-values of less than 0.05 have been considered statistically significant.

## Results

The results showed that 69.2% of the 120 participants were female, while 43.3% were between 26 and 35 years of age. The work experience of 36.6% of nurses was between 5 and 10 years. Other details related to the demographic characteristics of participants are described in Table 1.

**Table 1:** Demographic characteristics of participants (*n* = 120)

Variables		Frequency (percentage)
Age	≤ 25 years	36 (30)
	26–35 years	52 (43.3)
	≥ 36 years	32 (26.7)
Sex	Female	83 (69.2)
	Male	37 (30.8)
Education	Bachelor	107 (89.2)
	Postgraduate	13 (10.8)
Work experience	≤ 5 years	41 (34.2)
	5–10 years	44 (36.6)
	≥ 10 years	35 (29.2)
Employment status	Tarhi^a^	21 (17.5)
	Gharardad^b^	64 (53.3)
	Rasmi^c^	35 (29.2)

^a^Who undertakes

^b^Contract Employment

^c^Permanent Employment

**Fig. 1:**
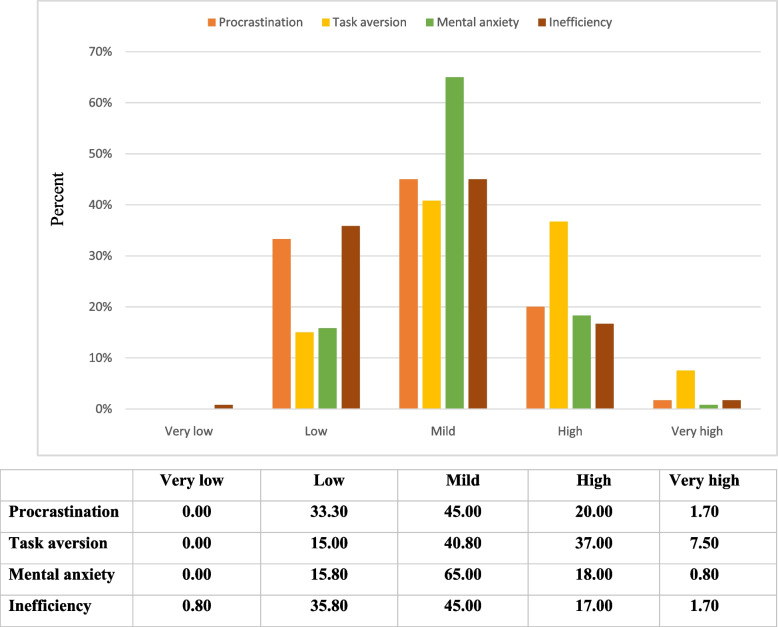
The profile of the participants in terms of the severity of the different aspects of procrastination

In addition, 37% of the participants showed high and very high levels of procrastination. “Task aversion” was observed in 44.2%, while “Mental anxiety” was observed in 21.1%, and "Inefficiency" was also observed in 18.4% of the participants considered to be high. The results of the severity of procrastination are shown in Fig. 1.

The determination of procrastination based on age, work experience, sex, employment, and educational status was assessed in data normality (*p* = 0.147) by analysis of variance (ANOVA). According to Table 2, procrastination had a significant relationship with the age of the participants. It is concluded that the average procrastination level was lower for older people, and the difference was substantial (*p* = 0.013). In addition, there was less procrastination among married people. (*p* = 0.02).

**Table 2:** Effect of variables on aspects of procrastination

		**Inefficiency (80 scores) mean ± SD**	**Mental anxiety (25 scores) mean ± SD**	**Task aversion (20 scores) mean ± SD**	**Procrastination (125 scores) mean ± SD**
**Work experience**	< 5 year	48.17 ± 10.57	15.95 ± 2.5	13.85 ± 2.6	77.97 ± 14.7
	5–10 year	47.35 ± 12.5	15.67 ± 2.9	13.65 ± 3.5	76.7 ± 18.2
	10 year ≤	40.37 ± 11.6	14.28 ± 2.4	11.77 ± 3.09	66.4 ± 17.3
	Total	45.39 ± 12.13	15.3 ± 2.7	13.13 ± 3.23	73.84 ± 17.3
	**F**	5.04	4.32	5.07	5.4
	***p*-value**	0.008	0.016	0.008	0.006
**Sex**	Male	41.46 ± 10.5	14.97 ± 2.4	12.05 ± 2.9	68.5 ± 14.8
	Female	47.14 ± 12.5	15.5 ± 2.8	13.6 ± 3.3	76.2 ± 17.9
	T	- 2.42	-0.954	-2.48	-2.3
	***p*-value**	0.017	0.343	0.015	0.023
**Education**	Bachelor	46.48 ± 11.8	15.6 ± 2.6	13.4 ± 3.09	75.5 ± 16.57
	Postgraduate	11.65 ± 3.4	13.5 ± 3.2	11 ± 3.6	62 ± 18
	T	2.51	2.6	2.5	2.65
	***p*-value**	0.013	0.011	0.013	0.009
**Employment status**	Tarhi^a^	47.1 ± 11.7	15.95 ± 2.7	13.4 ± 2.8	76.5 ± 16.3
	Gharardad^b^	46.8 ± 9.3	15.7 ± 1.9	13.5 ± 2.8	76 ± 12.9
	Rasmi^c^	42.1 ± 12.35	14.95 ± 2.73	12.8 ± 3.2	71.95 ± 17.63
	**F**	3.34	2.88	4.05	3.68
	***p*-value**	0.022	0.039	0.009	0.014

^a^Who undertakes

^b^Contract Employment

cPermanent Employment

It is noted that the average procrastination was lower in association with high work experience, and there was a significant difference (*p* = 0,006). The average procrastination score for women was also higher (*p* = 0.023).

In addition, the average procrastination score for graduates was higher than for postgraduates (*p* = 0,009). The average procrastination score for government employees was lower than for formal workers (*p* = 0.014). According to the Friedman test, there was a significant difference between the dimensions of procrastination. The highest and lowest average scores were allocated to "Inefficiency" and "Mental anxiety" dimensions, respectively (Table 3).

**Table 3:** Correlation between different aspects of procrastination

		**procrastination**	**Task aversion**	**Mental anxiety**	**Inefficiency**
**Inefficiency**	**r**	0.99	0.866	0.818	1
	**P**	0.000	0.000	0.000	
**Mental anxiety**	**r**	0.871	0.755	1	
	**P**	0.000	0.000		
**Task aversion**	**r**	0.911	1		
	**P**	0.000			

Also, three main reasons for procrastination were: "I believe that if I perform my duties in time, I will immediately receive another duty, "Although I am sincere in doing my duties, there is not enough respect for me" and "It is hard for me to give priority to my duties".

## Discussion

The main objective of this research was to evaluate the level of procrastination among nurses working in educational surgical wards and ICUs. The results showed that most participants had a mild level of procrastination.

Due to their health conditions and life-threatening situation, patients hospitalized in intensive care and surgery wards are entirely dependent on and need to receive more professional care. This dependency causes nurses to spend more time caring for the patient. Meanwhile, other care measures, such as recording a detailed nursing report, following up on para-clinical procedures, and recording treatment documentation, take up more time during the shift. Also, considering the lack of staffing, the standard ratio of nurses to patients has not been observed in these units. Since in these departments, nurses have more contact with patients, and due to the lack of human resources and the conditions of patients, they are under more severe stress, which has caused mental turmoil in them and has had consequences such as inefficiency and mental anxiety. In addition, forced overtime hours, the heavy workload during the shift, and not receiving encouragement commensurate with their service can reduce motivation and increase task aversion among nurses.

This finding was consistent with the study examining procrastination and predictor variables, which found that half of the participants tended to procrastinate [19]. The result of several studies showed that most participants tended to have moderate to severe procrastination [19, 20, 33]. This issue, in turn, would have irreparable effects on academic, occupational, and personal performance, as postponement is associated with many highly reflective psychosocial health problems, dissatisfaction, time-management inefficiencies, and self-doubt.

Evidence indicates that factors such as occupational stress, fatigue, and lack of psychological compatibility with the profession lead to work delays [34]. Therefore, if there is a tendency for a moderate or high degree of procrastination to be seen in nurses, there should be evidence of an adverse individual and occupational situation.

It is concluded that the average procrastination is lower in older participants. In some studies, procrastination has not been associated with age [21, 22]. The study reported that variables such as age and performance range, including organizing and working memory, significantly predict procrastination, and among these factors, age is the most critical factor [17]. This finding is consistent with the study which examined the relationship between procrastination with demographic characteristics and distress and satisfaction. It concluded that younger people tended to procrastinate more [35]. As adults grow older, their values and skills, such as time management, targeting, etc., increase and mature. As a result of this phenomenon, their tendency to procrastinate is reduced directly, and this result is justifiable. It also has a significant and negative impact on the performance of individuals, so some actions are needed to mitigate adverse outcomes [20].

Similarly, in this study, the level of procrastination decreased while work experience increased. In a study with improved work experience, procrastination scores for nurses and midwives decreased, but this relationship was not statistically significant [22]. In another study, procrastination had a meaningful negative relationship with work experience [36]. It was found that procrastination in older people with social and work activities, is related to variables such as anxiety and stress, life satisfaction in its various dimensions, and so on [37]. On the other hand, neurobiological development is in progress with puberty [6]. Thus, as personal growth, during the process of evolution, and in particular in cognitive development, the ability to self-control develops. Which, in turn, can lead to a decrease in a person's tendency to procrastinate.

The result indicated less procrastination among married people, as confirmed by various studies [6, 35, 37]. It has been shown that people who feel more lonely are more likely to procrastinate [18]. In contrast, the results of a study between nurses and midwives indicated no relationship between marital status and procrastination [22]. Overall, educated, married, employed, and living in developed countries are less procrastinated [37]. In such a situation, people's quality of life is very high, and they suffer less stress.

The relationship between sex and procrastination has been repeatedly investigated, and shown that procrastination is significantly higher in males [6, 37]. This finding is justified by men's personality and psychological characteristics, higher levels of impulsiveness, and lower levels of self-control, which are essential components of postponing [6]. While the current study has had a controversial outcome, more procrastination has been seen in women. Perhaps a different result would have been achieved with more participants.

Despite what is mentioned, some studies show that the procrastination rate does not vary from one sex to another [19, 20]. A survey on procrastination and its impact on student's academic performance has shown that there is procrastination among participants, irrespective of gender [20].

In addition, the average procrastination score for graduates was higher than for postgraduates. The relationship between the level of education and postponing has been examined in several studies, and it has been noted that procrastinating in lower education is higher [6, 35, 37]. The inability of individuals to achieve academic achievements results from a weakness in self-regulation skills, which, in turn, has a direct relationship with procrastination, so this result is justifiable. The average procrastination score for permanent employees (Rasmi) was lower than for the others. These nurses are often staff with a high level of work experience who understand the importance of timely care and have a low level of procrastination.

Based on the average score, “Task aversion” was the most crucial reason for procrastination, while “Mental anxiety” and “Inefficiency” were the second and third reasons for procrastination. Researchers have found that procrastination is directly related to self-determined motivation. As the level of self-determined motivation decreases, the level of procrastination increases [10]. However, nurses considered "Inefficiency" to be the most essential reason for procrastination, while "Mental Anxiety" and "Task Aversion" were placed in the following rankings, respectively. A study showed an inverse and significant relationship between the amount of procrastination and the nurses' responsibility in the ICUs. There was also a significant association between procrastination and the motivation of nurses [1]. Self-confidence affects people's level of procrastination. People who neglect their duties have low self-confidence and are procrastinating or avoiding tasks, so they also try to justify their poor performance by apologizing for self-defense. In addition, a high level of self-esteem predicts high self-efficacy, and as both factors (self-confidence and high self-esteem) increase, procrastination decreases [38].

A study examined the relationship between obsessive–compulsive and cognitive beliefs and found direct and indirect relationships between these factors and negligence. Mental confidence was directly related to procrastination. Besides, cognitive self-consciousness had a reverse relationship with procrastination. Obsessive and mental beliefs must therefore be controlled to reduce this behavior. Control and formation of metacognitive beliefs can also be effectively reduce compulsive behavior [39]. Psychologically, improving their quality will be effective in reducing procrastination. Evidence suggests that time management reduces procrastination and increases individual abilities [8]. In addition, the determination of experimental objectives and achievements makes it possible for those who procrastinate to perform their duties on time. As people understand their obligations, their self-efficiency, self-approval, and performance are improved. Thereby the likelihood of procrastination is reduced, then work is completed.

It also is held that the delay concerns individual values. When valuable things, like success in work, are aligned with an individual's values, people tend to be less willing to procrastinate [10]. Procrastination causes unpleasant consequences for individuals and organizations, including loss of performance and reduced productivity, dissatisfaction, reduced employee motivation, and a negative attitude toward the organization.

## Recommendations of the study

Since procrastination is accompanied by psychiatric and behavioral disorders [40], further studies in this area are recommended to provide appropriate treatment if identified. On the other hand, taking into account the importance of the coherence of individual and organizational values that lead to procrastination control, management action must take to motivate and improve the professional environment. Therefore, it is suggested that more extensive research assesses health workers' procrastination status and other influential psychological variables.

## Limitations

There are some limitations to this study that should be considered when interpreting the results. Although the sampling was done in two super-specialized and specialized teaching hospitals in the metropolis, which have several surgeries and intensive care units, the number of samples and the research environment are considered limitations of the study. It makes difficult the generalizability of the results. Considering the importance of the topic and understanding the level of procrastination among treatment staff, the research would be done in hospitals and different medical wards.

It also mentioned the influence of environmental, personal factors, and mental and psychological nurses' conditions when completing the self-report questionnaire and the results obtained, which were beyond the researcher's control.

## Conclusion

As far as the results of this study are concerned, procrastination in nurses was moderate. The correlation between procrastination and mental health problems indicates that psychological problems, such as the inability to self-regulate and self-perceive, can lead to consequences such as behavioral disorders and professional tensions between nurses. It means that the objectives of the health system are in jeopardy. Furthermore, the quality of nursing care is significantly reduced, and cooperation and team performance is disrupted, which will be detrimental to the safety of patients. Results indicate the need for motivational and managerial intervention. Because procrastination causes unpleasant consequences for the organization, including loss of performance, reduced productivity and quality of care, dissatisfaction, negative attitude, reduced motivation for nurses, and irreparable harm to the patients.

## Data Availability

The data that support the findings of this study are available on request from the corresponding author.
